# Hyperphosphorylation of DegU cancels CcpA-dependent catabolite repression of *rocG* in *Bacillus subtilis*

**DOI:** 10.1186/s12866-015-0373-0

**Published:** 2015-02-22

**Authors:** Kosei Tanaka, Kana Iwasaki, Takuya Morimoto, Takatsugu Matsuse, Tomohisa Hasunuma, Shinji Takenaka, Onuma Chumsakul, Shu Ishikawa, Naotake Ogasawara, Ken-ichi Yoshida

**Affiliations:** Organization of Advanced Science and Technology, Kobe University, Kobe, Hyogo Japan; Department of Agrobioscience, Kobe University, Kobe, Hyogo Japan; Biological Science Laboratories, Kao Corporation, Haga, Tochigi Japan; Graduate School of Biological Sciences, Nara Institute of Science and Technology, Ikoma, Nara Japan

**Keywords:** *Bacillus subtilis*, Two-component regulatory system, Catabolite repression, Transcription, Metabolites

## Abstract

**Background:**

The two-component regulatory system, involving the histidine sensor kinase DegS and response regulator DegU, plays an important role to control various cell processes in the transition phase of *Bacillus subtilis*. The *degU32* allele in strain 1A95 is characterized by the accumulation of phosphorylated form of DegU (DegU-P).

**Results:**

Growing 1A95 cells elevated the pH of soytone-based medium more than the parental strain 168 after the onset of the transition phase. The *rocG* gene encodes a catabolic glutamate dehydrogenase that catalyzes one of the main ammonia-releasing reactions. Inactivation of *rocG* abolished 1A95-mediated increases in the pH of growth media. Thus, transcription of the *rocG* locus was examined, and a novel 3.7-kb transcript covering *sivA*, *rocG*, and *rocA* was found in 1A95 but not 168 cells. Increased intracellular fructose 1,6-bisphosphate (FBP) levels are known to activate the HPr kinase HPrK, and to induce formation of the P-Ser-HPr/CcpA complex, which binds to catabolite responsive elements (*cre*) and exerts CcpA-dependent catabolite repression. A putative *cre* found within the intergenic region between *sivA* and *rocG*, and inactivation of *ccpA* led to creation of the 3.7-kb transcript in 168 cells. Analyses of intermediates in central carbon metabolism revealed that intracellular FBP levels were lowered earlier in 1A95 than in 168 cells. A genome wide transcriptome analysis comparing 1A95 and 168 cells suggested similar events occurring in other catabolite repressive loci involving induction of *lctE* encoding lactate dehydrogenase.

**Conclusions:**

Under physiological conditions the 3.7-kb *rocG* transcript may be tightly controlled by a roadblock mechanism involving P-Ser-HPr/CcpA in 168 cells, while in 1A95 cells abolished repression of the 3.7-kb transcript. Accumulation of DegU-P in 1A95 affects central carbon metabolism involving *lctE* enhanced by unknown mechanisms, downregulates FBP levels earlier, and inactivates HPrK to allow the 3.7-kb transcription, and thus similar events may occur in other catabolite repressive loci.

**Electronic supplementary material:**

The online version of this article (doi:10.1186/s12866-015-0373-0) contains supplementary material, which is available to authorized users.

## Background

Bacteria possess two-component regulatory systems comprising environmental sensor histidine kinases and cognate response regulators that are involved in adaptation to various chemical, physical, and physiological stimuli [1]. Following stimulation, autophosphorylation of specific sensor histidine kinases leads to transfer of a phosphoryl group to cognate transcriptional response regulators that control respective target genes.

In *Bacillus subtilis*, the DegS–DegU two-component regulatory system controls various processes during the transition from exponential to stationary growth phases, including the expression of extracellular degradative enzymes and late competence genes [2]. The phosphorylated form of DegU (DegU-P) induces a number of genes, whereas the unphosphorylated form also stimulates transcription of comK, which encodes the competence transcription factor [3,4]. In addition, DegU-P negatively regulates genes of the sigma factor SigD regulon, which are involved in motility, chemotaxis, and autolysin production [2,5,6]. In addition, the DegS–DegU system is modulated by two regulatory genes, *degQ* and *degR*, which encode small polypeptides of 46 and 60 amino acids, respectively [7]. In wild-type strains, DegQ enhances phosphorylation of DegU [8], whereas DegR protects DegU-P from dephosphorylation [9]. In contrast in the standard laboratory strain 168, due to a mutation in the −10 region (T-10 to C) of its cognate promoter, *degQ* is not efficiently transcribed, and DegU-P does not accumulate as in wild-type strains [10].

In the previous studies, mutations in DegU led to overproduction of extracellular degradative enzymes and correlated with the loss of natural competence for DNA uptake, the lack of flagella synthesis, filamentous morphology, and higher sporulation efficiency in the presence of glucose [11-13]. Among these mutations, the allele *degU32* in strain 1A95 has an amino acid substitution at position 12 [14]. This mutation increases the stability of DegU-P sevenfold, and amplifies DegU-P dependent gene expression even with the genetic background of strain 168 [13]. Extracellular proteomic studies of the *degU32* mutant indicated that 13 extracellular enzymes are overproduced and that eight proteins of motility and cell-wall turnover were significantly downregulated, including five SigD-dependent proteins [15]. The subsequent transcriptome analysis confirmed similar induction and repression of the known DegU-P and DegU dependent genes, respectively [16].

Bacterial carbon catabolite repression (CCR) is a global regulatory mechanism that occurs in the presence of a preferred carbon source and represses genes involved in metabolism of other minor carbon sources, coordinating metabolic responses to efficient carbon and energy sources. In *B. subtilis*, CCR occurs primarily at the transcription level and involves phosphorylation of CcpA and HPr at the Ser-46 residue (P-Ser-HPr) [17]. Preferred carbon sources such as glucose are readily converted into glycolytic intermediates, including fructose-1,6-bisphosphate (FBP), and the accumulation of intracellular FBP stimulates HPr kinase/phosphatase, which phosphorylates HPr to P-Ser-HPr. CcpA is a member of the LacI/GalR family of transcriptional regulators, which are synthesized constitutively irrespective of the presence or absence of repressing sugars. The CcpA/P-Ser-HPr complex is formed only in response to P-Ser-HPr, and exerts site-specific DNA-binding activity with 14-bp cis-acting palindromic sequences that are known as catabolite responsive elements (*cre*). Because DNA binding of CcpA/P-Ser-HPr interferes with both initiation and elongation of target gene transcription under CCR, CcpA/P-Ser-HPr functions as a classical repressor and a transcriptional roadblock. In contrast, HPr originally functions as the energy coupling protein of the phosphotransferase system (PTS), which mediates carbohydrate transport following phosphorylation of the His-15 residue.

*B. subtilis* expresses the two glutamate dehydrogenase genes *gudB* and *rocG*. In the standard laboratory strain 168 and its derivatives, *gudB* is a cryptic gene with a short repeat within the coding region, whereas in the wild-type *B. subtilis* strains this gene encodes an intact enzyme [18]. Potentially, the ancestral *gudB* gene became inactive during domestication under laboratory conditions. In contrast, RocG functions as an active catabolic glutamate dehydrogenase and is induced in the presence of arginine, ornithine, or proline following transcriptional activation of the SigL-dependent promoter and enhancement by the transcription factor RocR [19]. RocG is also regulated by CcpA-dependent CCR, involving a *cre* situated just downstream of the *rocG* promoter [20]. Moreover, RocG is involved in ammonia-releasing reactions during the production of the Japanese food natto, which is produced by fermentation of soybeans using strains of *B. subtilis* natto that are equivalent to wild-type *B. subtilis* strains [18].

As described above, 1A95 cells carry the *degU32* mutation on the genetic background of the laboratory strain 168. In the present study, we noticed that the pH of soytone-based medium significantly increased during growth of 1A95 cells after the onset of the transition phase. Thus, we speculated that this change in pH reflects ammonia release following glutamate degradation by RocG. Subsequent investigations of transcription in the *rocG* locus revealed a novel transcript in 1A95 cells. Further experiments indicated that hyperphosphorylation of DegU may proactively abolish CcpA-dependent CCR, modulating the regulatory network involving intracellular metabolites and phosphorylation of HPr.

## Results

### The *degU32* mutation led to elevated pH of growth medium following activation of *rocG* encoding glutamate dehydrogenase

**Figure 1:**
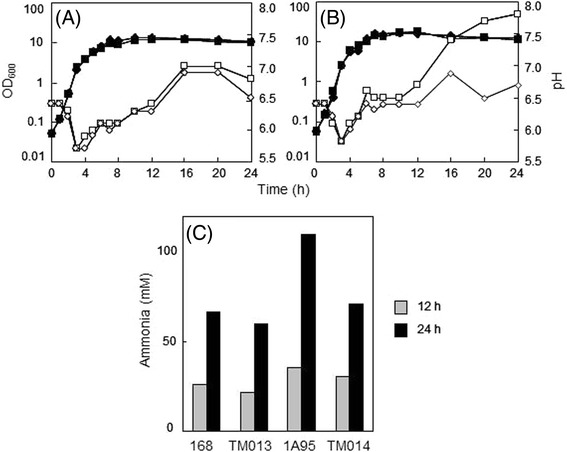
**Involvement of rocG in elevated pH and ammonia levels in the growth medium of strain 1A95 (degU32). (A)** Growth curves of *B. subtilis* strains 168 (closed squares) and TM013 (*rocG*::*cat*, closed diamonds) in the soytone–glucose medium; pH values are indicated for 168 (open squares) and TM013 (open diamonds) cultures. **(B)** Growth curves of strains 1A95 (*degU32*, closed squares) and TM014 (*degU32 rocG*::*cat*, closed diamonds) in the soytone–glucose medium; pH values are indicated for 1A95 (open squares) and TM014 (open diamonds) cultures. **(C)** Levels of ammonia in media at 12 (gray bars) and 24 (black bars) h after inoculation.

During growth of *B. subtilis* strains in soytone–glucose medium, pH levels decreased to around 5.5 prior to transition from the logarithmic to the stationary phase, and then increased during the stationary phase (Figure 1). However, pH levels gradually increased to around 6.5 and no higher than 7.0 during growth of the laboratory standard strain 168, whereas the 1A95 (*degU32*) strain significantly elevated pH to almost 8.0.

*rocG* encoding glutamate dehydrogenase mediates ammonia-releasing reactions during secondary natto fermentation [18]. Thus, we suspected that ammonia may contribute to increases in the pH of growth media, and observed ammonia accumulation in the culture medium of 1A95 cells (Figure 1). Inactivation of *rocG* in the 168 strain only had a slight effect on pH and ammonia accumulation (Figure 1A and C, strain TM013). However, inactivation of *rocG* in 1A95 cells prevented increases in pH and accumulation of ammonium, which remained at similar levels to those observed in 168 cells (Figure 1B and C, strain TM014). These data indicate that *degU32* contributes to increases in pH, potentially by enhancing RocG-mediated release of ammonia.

### Cells expressing *degU32* produced a novel 3.7-kb transcript containing *rocG*

**Figure 2:**
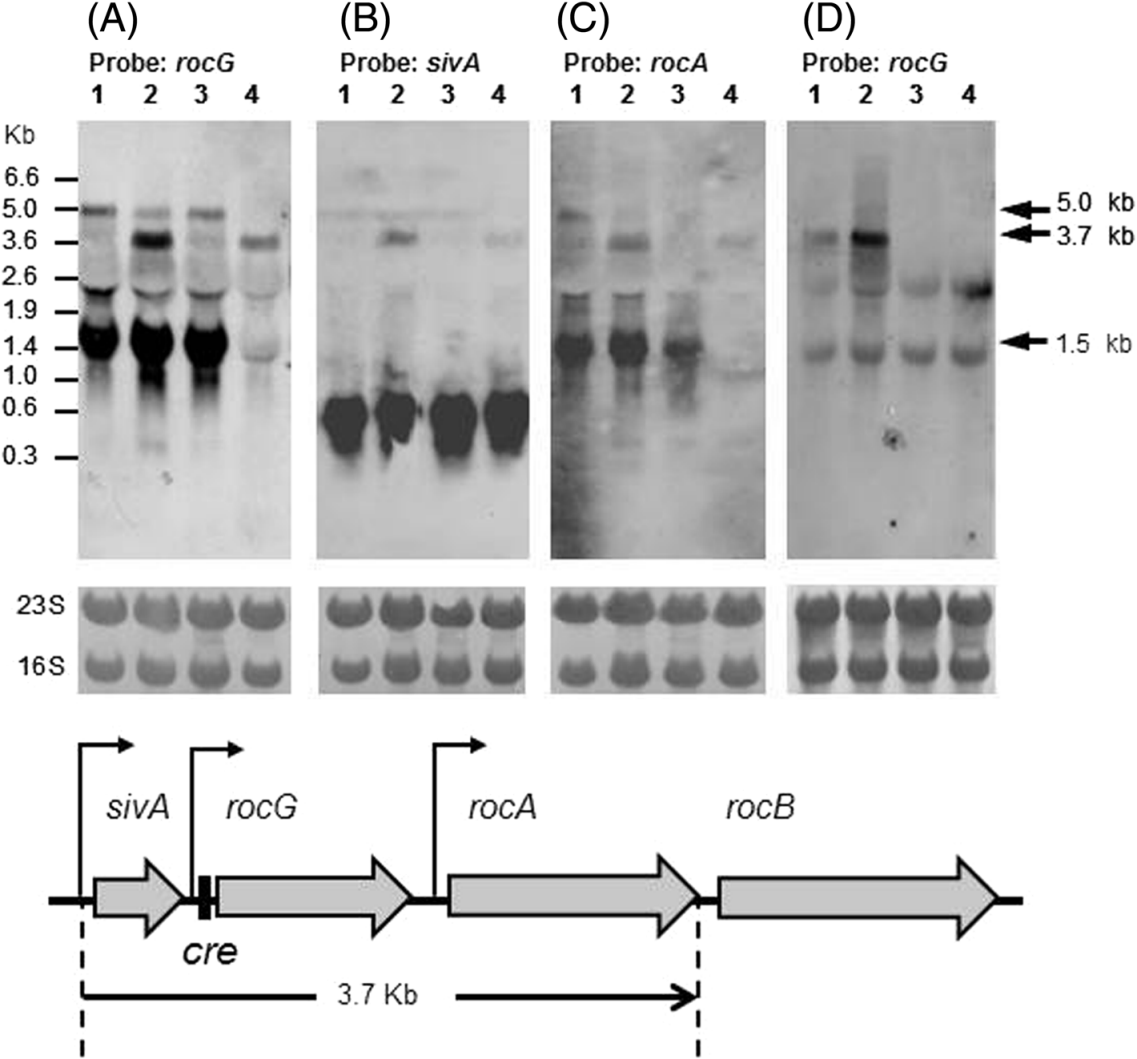
**Northern blotting analyses of transcripts containing** ***rocG*** **,** ***sivA*** **, and** ***rocA.*** RNAs were prepared from 168 (lane 1), 1A95 (*degU32*, lane 2), TM015 (*degU32*::*cat*, lane 3), and TM016 (*degU32 rocR*::*kan*, lane 4) cells for northern blotting analyses using probes for *rocG* **(A)**, *sivA* **(B)**, and *rocA* **(C)** transcripts under low-stringency conditions. **(D)** RNAs were prepared from 1A95 (*degU32*, lane 1), TM016 (*degU32 rocR*::*kan*, lane 2), TM017 (*sivA*::pMutin2, lane 3), and TM018 (*degU32 sivA*::pMutin2, lane 4) cells for northern blotting analyses using probes for *rocG* transcripts under high-stringency conditions; rRNAs (23S and 16S) on membranes were visualized as a loading control using methylene blue staining. Gene organization of the *rocG* locus is shown at the bottom.

Total RNAs were extracted from cells during the transition from logarithmic to stationary phases, just prior to pH increases (Figure 1B). In subsequent northern blotting analyses (Figure 2), three mRNA species containing *rocG* were detected in 1A95 cells under low-stringency conditions, with sizes of 5.0, 3.7, and 1.5 kb (Figure 2A). Control of *rocG* by RocR reportedly activates the SigL-dependent promoters for *rocABC*, *rocDEF*, and *rocG* in the presence of arginine metabolites such as citrulline and ornithine [19-21]. Both 5.0- and 1.5-kb transcripts were detected in 168 cells, but were not present in TM016 cells (*degU32 rocR*::*kan*). These observations indicate probable dependence of these two transcripts on *rocR* but not on *degU32*, although further experiments were not performed on these transcripts because they were not detected under high-stringency conditions (see below). The signals for the 1.5-kb transcript were rather exaggerated probably due to a synergistic effect of non-specific hybridization to 16S rRNA under the low-stringency conditions. In contrast, appearance of the 3.7-kb transcript depended solely on *degU32* in 1A95 and TM016 cells and was not present in strains 168 and TM015 (*degU32*::*cat*), indicating that this novel 3.7-kb transcript may enhance the function of RocG in 1A95 cells.

**Figure 3:**
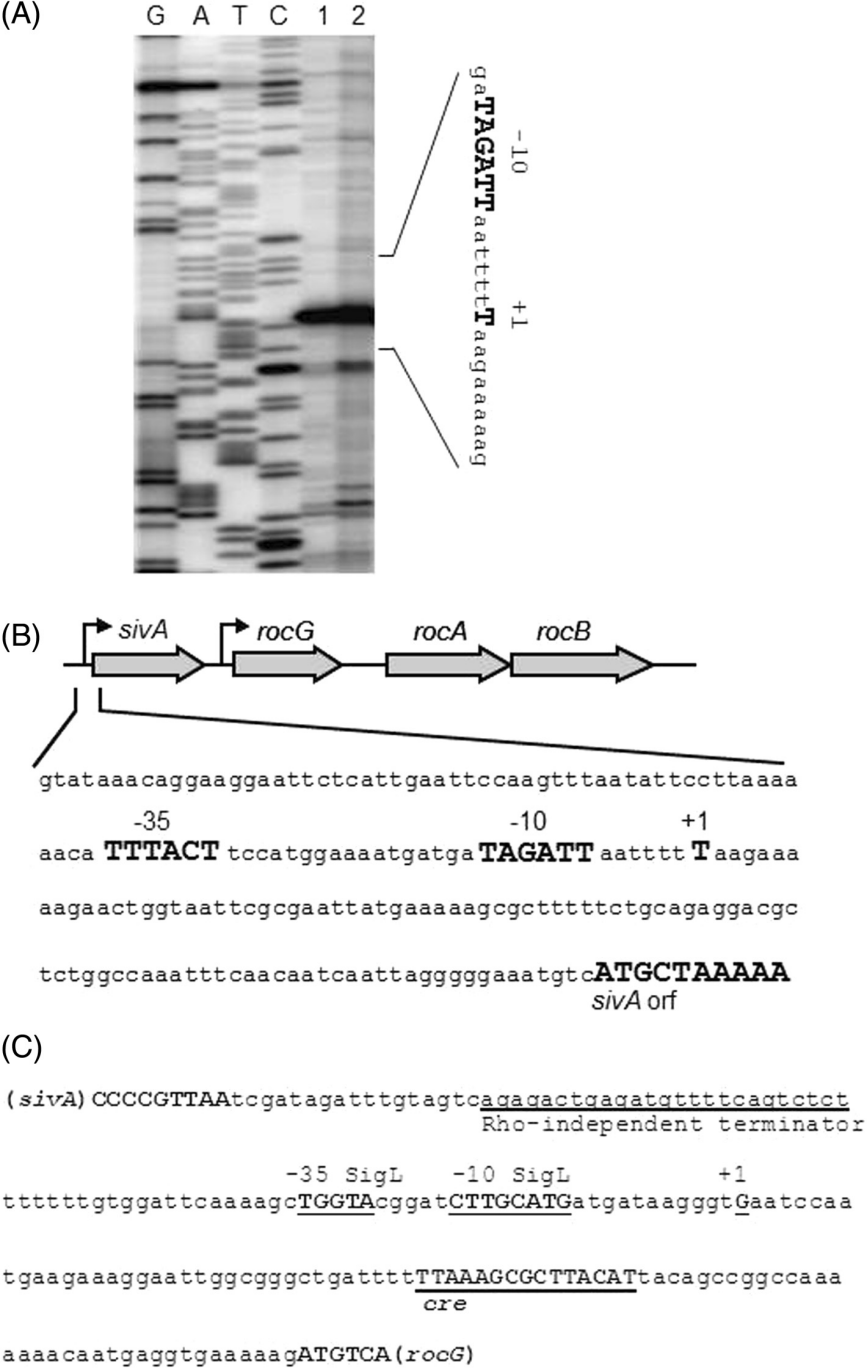
**The promoter and terminator regions of** ***sivA.*** **(A)** Primer extension analyses of the *sivA* promoter region; RNAs were prepared from 168 (lane 1) and 1A95 (*degU32*, lane 2) cells, and were reverse transcribed to generate cDNAs corresponding to the 5′-terminus of *sivA* transcripts. Lanes G, A, T, and C contained dideoxy sequencing ladders. The partial nucleotide sequence of the coding strand is shown on the right side, where the putative −10 region and the transcription start site (+1) are shown in enlarged uppercase characters. **(B)** Schematic presentation of the *sivA* promoter region. The nucleotide sequence of the upper strand of the promoter region is shown, and putative −35 and −10 regions, the transcription start site (+1), and the beginning of the *sivA* open reading frame are shown with enlarged uppercase characters. **(C)** Nucleotide sequence of the intergenic region between *sivA* and *rocG*. The C-terminal end of *sivA*, the Rho-independent terminator, SigL-dependent *rocG* promoter (+35 and −10 regions), its transcription start site (+1), the *cre* site, and the N-terminal end of *rocG* are indicated.

The 3.7-kb transcript was weakly but substantially detected under low-stringency conditions using either *sivA* (formerly *yweA*) or *rocA* probes in 1A95 and TM016 cells (Figure 2B and C), suggesting coverage of *rocG*, *sivA*, and *rocA*. A very strong 0.6-kb *sivA* transcript was observed in all four strains, suggesting transcription from a constitutive promoter upstream of *sivA* (Figure 2B). The probe for *rocA* detected another 1.5-kb transcript in 168, 1A95, and TM015 cells, but not in TM016 cells, suggesting dependence on *rocR* (Figure 2C). Even under high-stringency conditions, the 3.7-kb transcript was clearly detected using the *rocG* probe in 1A95 and TM016 cells, but was absent after disruption of *sivA* in TM018 cells (*degU32 sivA*::pMutin2; Figure 2D). The 5.0- and 1.5-kb *rocG* transcripts observed under low-stringency conditions (Figure 2A) were not detectable under high-stringency conditions in 1A95 cells (Figure 2D), suggesting that the 3.7-kb transcript could be the major *rocG* transcript in 1A95 cells and is dependent on *degU32*. Primer extension analyses revealed a transcription start site at 97 bases upstream of the translation start site of *sivA* (Figure 3). Thus, the corresponding promoter may comprise the −35 (TTTACT) and −10 (TAGATT) regions with a 17-bp spacer and may be a typical SigA-dependent one. The constitutive 0.6-kb transcript may be the main *sivA* transcript, and is produced independently of *degU32* or *rocR* (Figure 2B). Accordingly, the present northern analyses (Figure 2B) and a previous study indicate that transcription from the *sivA* promoter may be independent of both SigL and RocR [22].

### Control of the 3.7-kb rocG transcript by CcpA-dependent CCR

A previous study indicated that *rocG* is regulated by CcpA-dependent CCR, involving a putative *cre* site in the intergenic region between *sivA* and *rocG* [20]. Moreover, the *sivA* transcript may be read through to cover downstream *rocG* [22]. CcpA-dependent CCR involves not only repression of transcriptional initiation at promoter sites but also a roadblock mechanism that aborts ongoing transcription [17]. Along with previous results, the present data suggest that the 3.7-kb transcript is produced by transcription through the main 0.6-kb *sivA* and elongation to cover both *rocG* and *rocA*, and thus escapes CcpA-dependent CCR. Although a Rho-independent terminator (ΔG = −16.90 kcal/mol, Figure 3C) is present and may terminate transcription from the *sivA* promoter, it may allow some read through. Moreover, CcpA-dependent CCR may arrest the read via the roadblock mechanism in 168 cells but not in 1A95 cells. It was previously shown that *rocABC* composed a single 5.0-kb transcriptional unit depending on both SigL and RocR [23]. Another northern blotting analysis revealed that a *rocB* specific probe detected the 5.0-kb transcript but failed to detect the 3.7-kb one (Additional file 1: Figure S1). Recently an internal terminator was found just behind *rocA* [24,25], and this terminator could function in this case to generate the 3.7-kb transcript covering *sivA*, *rocG*, and *rocA*. The SigL-dependent *rocG* promoter and its corresponding transcription start site are located upstream of the *cre* site [26]. Therefore, the CcpA-dependent roadblock might also control SigL-dependent *rocG* transcription.

**Figure 4:**
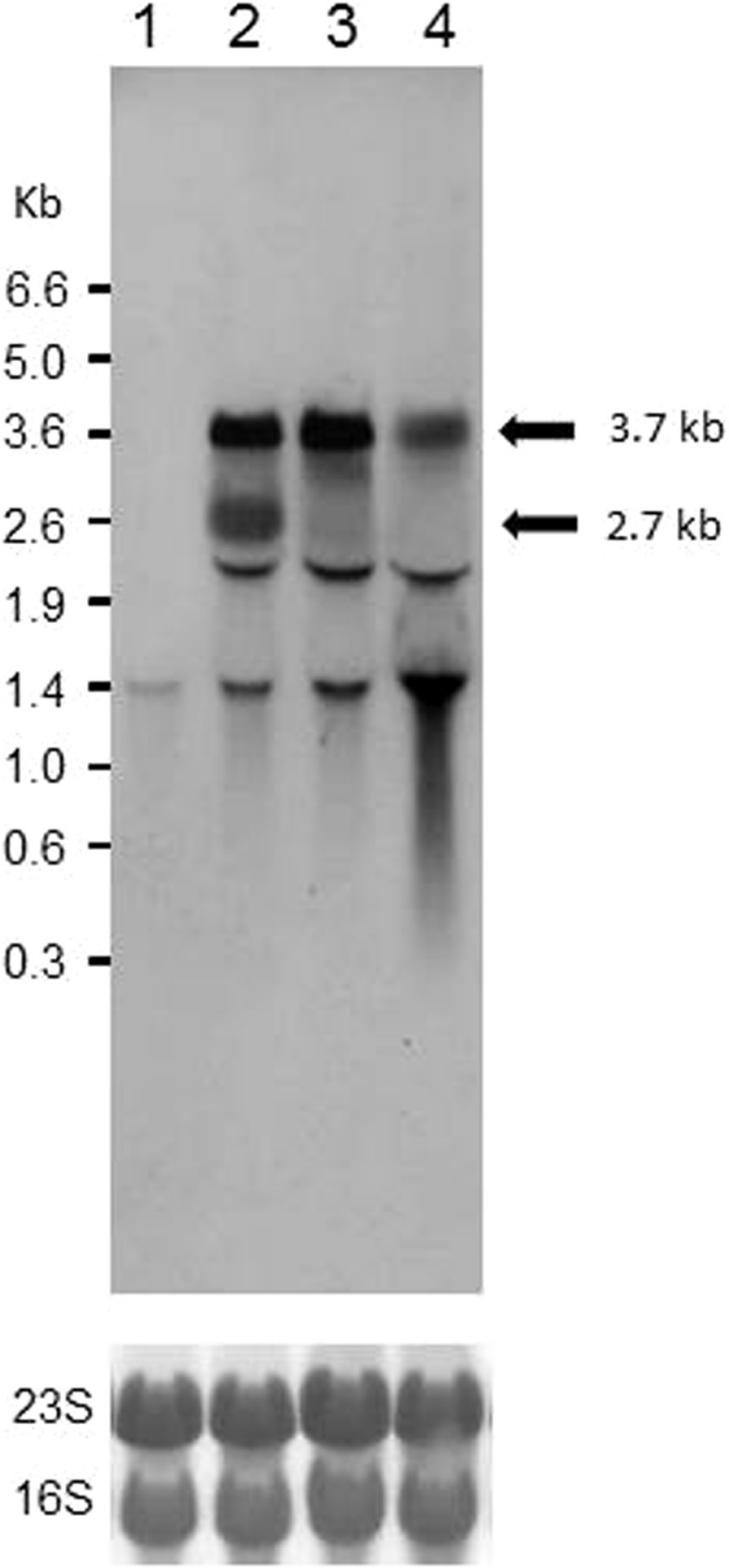
**Northern blotting analyses of** ***rocG*** **transcripts in the presence and absence of** ***ccpA.*** RNAs were prepared from 168 (lane 1), TM023 (*ccpA*::*neo*, lane 2), 1A95 (*degU32*, lane 3), and TM024 (*degU32 ccpA*::*neo*, lane 4) cells for northern blotting analyses using a probe for *rocG* transcripts under high-stringency conditions. Experiments were repeated independently at least three times with similar results, and representative data are shown. At the bottom, rRNAs (23S and 16S) were visualized as loading controls using methylene blue staining.

When *ccpA* was inactivated (*ccpA*::*neo*) in strain 168, the 3.7-kb transcript containing *rocG* was produced as in 1A95 cells (Figure 4; note that the 5.0- and 1.5-kb transcripts were not detected under high-stringency conditions). These observations indicated that the 3.7-kb transcript was repressed by CcpA-dependent CCR in 168 cells and was likely “induced” in the 1A95 (*degU32*) strain, allowing proactive abolition of the CcpA-dependent CCR roadblock at the *cre* in the intergenic region between *sivA* and *rocG* (Figure 3C). However, the *ccpA* mutation in 168 produced an additional 2.7-kb transcript that remains uncharacterized.

### Intracellular FBP levels were reduced earlier in *degU32* mutant cells due to enhanced expression of *lctE*

**Figure 5:**
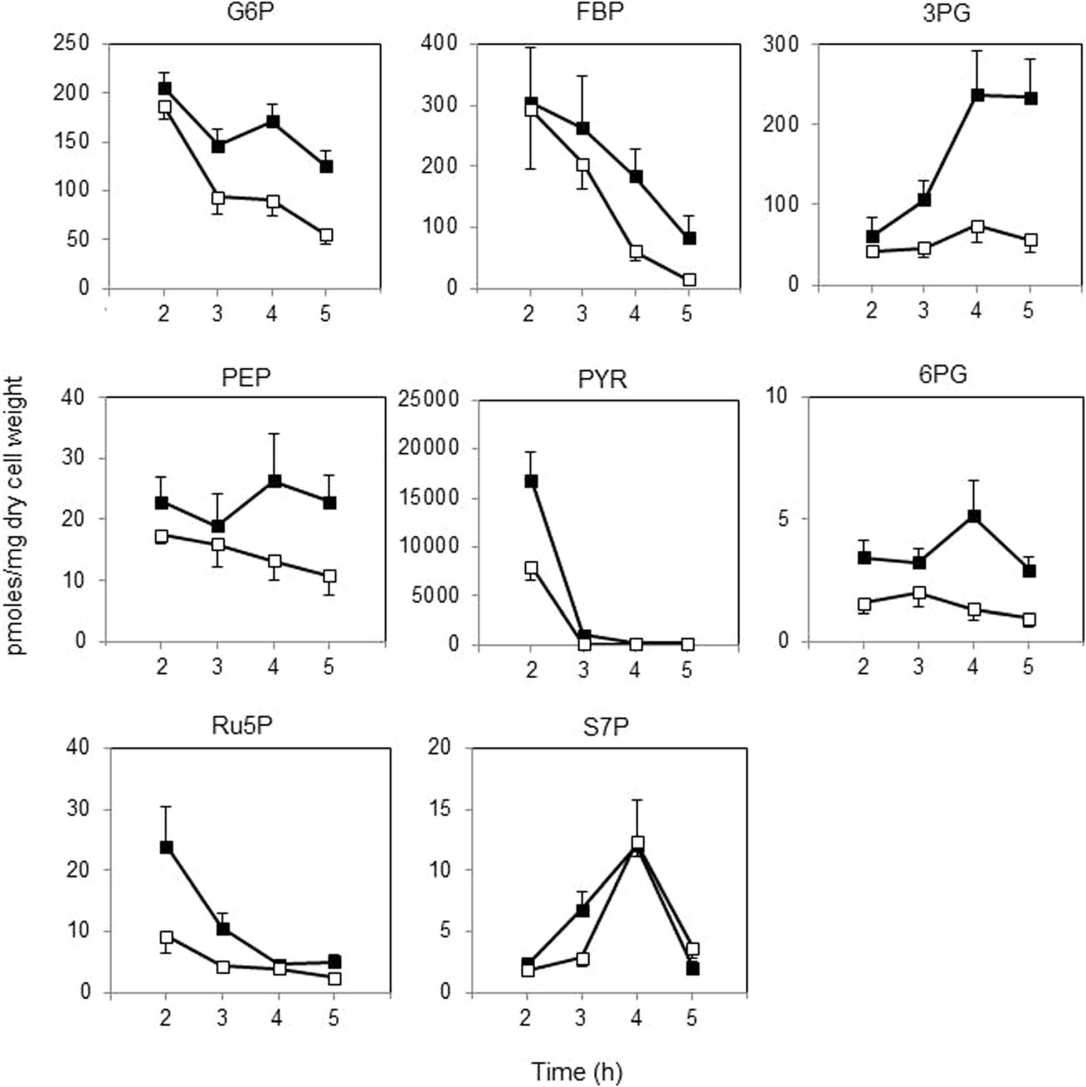
**Intracellular levels of central carbon metabolism intermediates.** Cells of 168 (closed squares) and 1A95 (*degU32*, open squares) strains were grown in the soytone–glucose medium and intracellular metabolites were extracted at the indicated time points after inoculation for CE–MS analyses. Experiments were repeated independently for three times, and the mean values are shown with standard deviation. Intermediates are abbreviated as follows: glucose 6-phosphate, G6P; fructose-1,6-bis-phosphate, FBP; 3-phosphoglyceric acid, 3PG; phosphoenolpyruvate, PEP; pyruvate, PYR; 6-phosphogluconate, 6PG; ribulose 5-phosphate, Ru5P; and sedoheptulose 7-phosphate, S7P.

To investigate abolition of CcpA-dependent CCR in 1A95 (*degU32*) cells, intracellular intermediates of central carbon metabolism were determined by capillary electrophoresis–mass spectrometry (CE–MS) analysis and compared with those in 168 cells (Figure 5). FBP levels decreased during the growth in both strains, and the decrease occurred faster in 1A95 cells. Glucose 6-phosphate (G6P) levels also decreased in 1A95. In contrast, 3-phosphoglyceric acid (3PG), phosphoenolpyruvate (PEP), and pyruvate (PYR) levels were consistently lower in 1A95 cells than in 168 cells. Thus, glycolysis might proceed more efficiently in 1A95 than in 168 cells, and the faster reduction in the FBP levels led to an earlier alleviation of CcpA-dependent CCR. On the other hand, in both strains, levels of intermediates of the pentose phosphate pathway, including 6-phosphogluconate (6PG), ribulose 5-phosphate (Ru5P), and sedoheptulose 7-phosphate (S7P), were generally lower than the glycolytic intermediates, such as G6P, FBP, and 3PG, nevertheless some significant effects of *degU32* were seen to lower the levels of intermediates in the pentose phosphate pathway. Potentially, glycolysis might consume glucose more efficiently in 1A95 cells, as reflected by lower levels of 6PG, Ru5P, and S7P.

**Figure 6:**
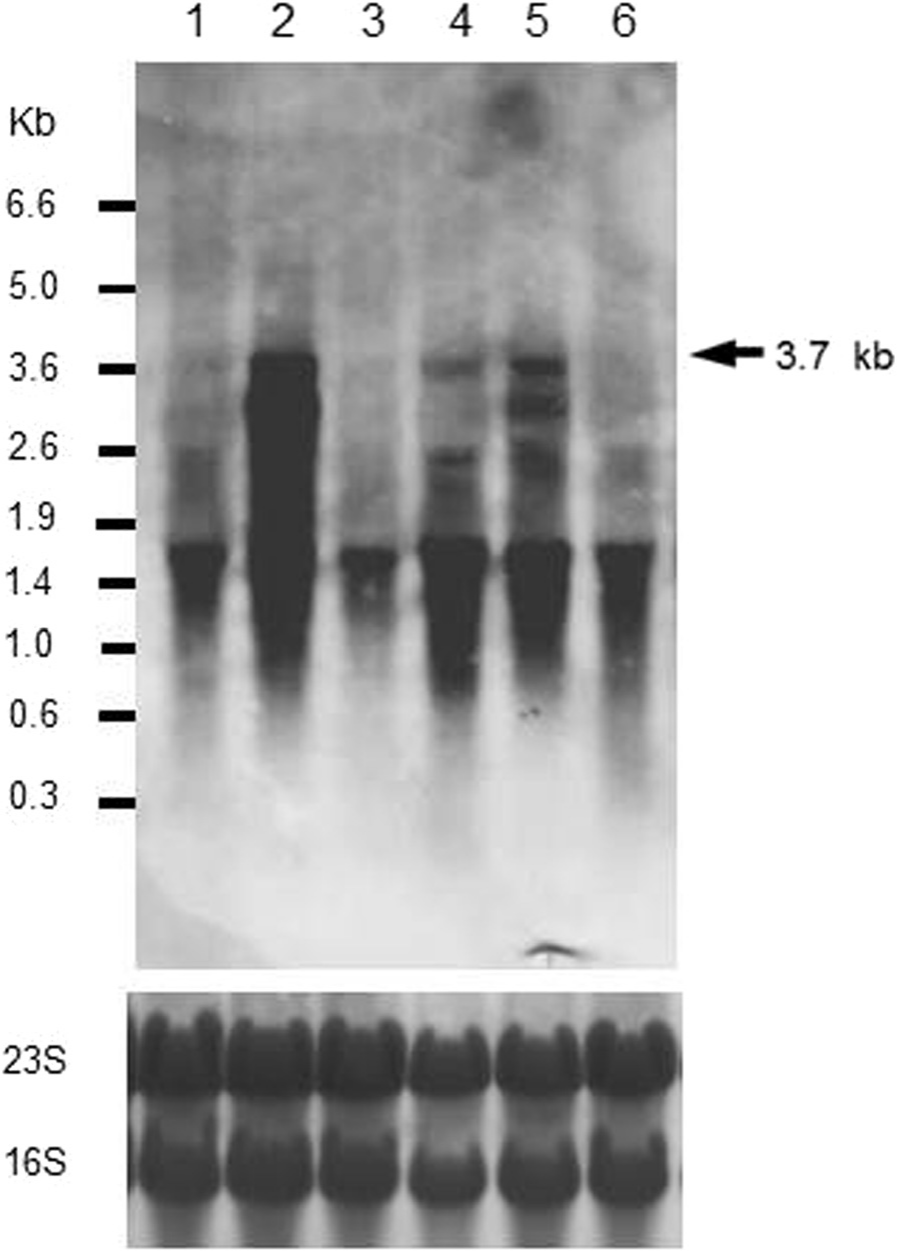
**Northern blotting analysis of** ***rocG*** **transcripts in the presence and absence of** ***lctE.*** RNAs were prepared from 168 (lane 1), TM023 (*ccpA*::*neo*, lane 2), MY02 (*lctE*::*spc*, lane 3), 1A95 (*degU32*, lane 4), TM024 (*degU32 ccpA*::*neo*, lane 5), and KI004 (*degU32 lctE*::*spc*, lane 6) cells for northern blotting analyses using probes for *rocG* transcripts under high-stringency conditions. Experiments were repeated independently at least three times with similar results, and representative data are shown. At the bottom, rRNAs (23S and 16S) were visualized as loading controls using methylene blue staining.

**Figure 7:**
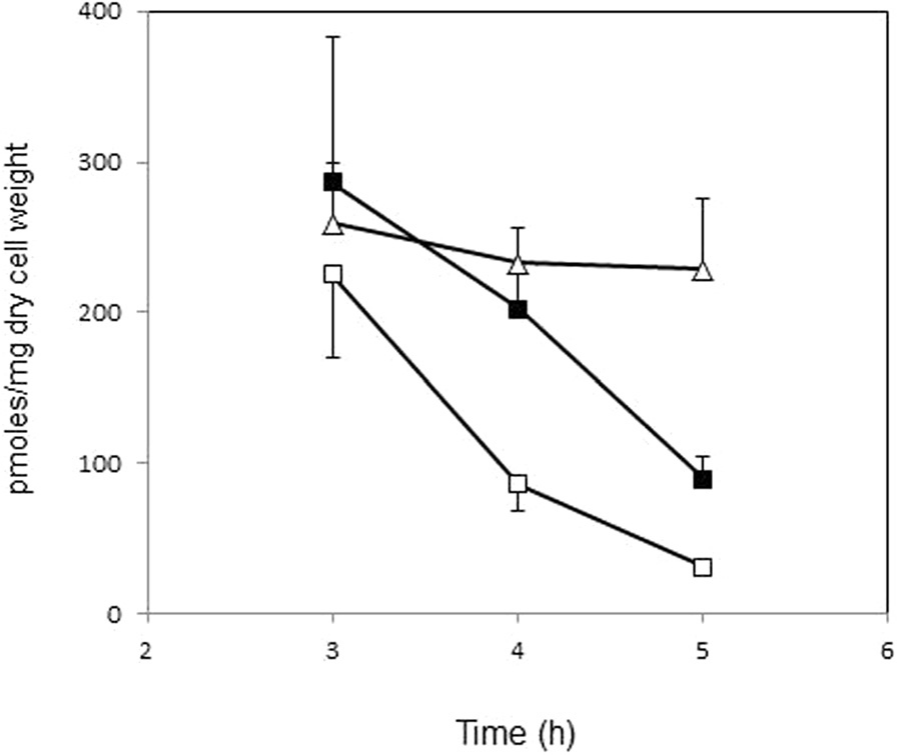
**Intracellular levels of FBP in the presence and absence of** ***lctE.*** Cells of 168 (closed squares), 1A95 (*degU32*, open squares), and KI004 (*degU32 lctE*::*spc*, open triangles) cells were grown in the soytone–glucose medium, and intracellular metabolites were extracted at the indicated time points and were analyzed using CE–MS. Experiments were repeated independently for three times, and the mean values are shown with standard deviation.

Transcriptome analyses were performed to compare 168 and 1A95 during transition from the logarithmic to the stationary phase. Among 25 selected genes of glycolysis, pentose phosphate, and branching fermentation pathways for lactate, acetoin, and acetate production, *lctE*, *ackA*, and *gntZ*, were enhanced by 5.44-, 5.86-, and 4.53-fold, respectively, in 1A95 cells compared with 168 cells (Table 1). *lctE* for lactate dehydrogenase was prominently expressed in 1A95 cells and its inactivation led to disappearance of the 3.7-kb transcript containing *rocG* (Figure 6) and maintenance of high intracellular FBP levels (Figure 7). Thus, *degU32* in 1A95 cells may enhance the expression of *lctE* through unknown mechanisms. Because PYR is the end product of glycolysis, rapid clearance of PYR due to its conversion into lactate by lactate dehydrogenase (LctE) might accelerate glycolysis, and lower intracellular FBP levels might cancel CcpA-dependent CCR earlier. *gntZ* for 6PG dehydrogenase is part of the *gntRKPZ* operon for gluconate catabolism [27], which is induced specifically in the presence of gluconate and repressed with glucose via CcpA-dependent CCR [28]. There is an additional promoter upstream of *gntZ* (within the *gntP* coding region), which allows transcription of *gntZ* independently [27]. Although this *gntZ*-specific transcription is not under CCR, it might be involved in the enhanced expression. *ackA* for acetate kinase is positively regulated by CcpA in the presence of glucose [29,30], involving either phosphorylated HPr or Crh [31]. Therefore, when FBP levels get lower, *ackA* was expected to be down regulated. But under the present conditions, *ackA* was induced probably due to CodY function as discussed below.

**Table 1:** **Transcriptome analyses of 168 and 1A95 cell** ^**a**^

**Gene** ^**b**^	**Signal in 168**	**Signal in 1A95**	**Ratio (1A95/168)**
ptsG	868.57	5437.79	6.26
*ackA*	392.94	2301.16	5.86
*lctE*	1031.06	5603.86	5.44
*gntZ*	209.03	947.25	4.53
sacP	194.83	680.86	3.49
dra	405.07	1210.64	2.99
yxkJ	339.75	991.7	2.92
resB	802.05	2072.13	2.58
rbsC	54.75	133.97	2.45
rocG	220.35	470.1	2.13
kdgA	87.12	161.59	1.85
*rpe*	657.61	1190.12	1.81
*pdhA*	2110.16	3796.04	1.80
rbsK	167.65	278.05	1.66
*pfkA*	2557.11	4158.71	1.63
rbsA	94.2	153.12	1.63
rbsR	178.33	280.61	1.57
*pyk*	2613.65	4060.8	1.55
licB	137.4	210.68	1.53
rbsD	55.17	83.13	1.51
ccpC	369.74	534.18	1.44
cydA	332.81	436.13	1.31
acuA	364.04	452.95	1.24
*tpiA*	3565.89	4427.84	1.24
*eno*	4240.4	5052.53	1.19
cccA	417.01	476.84	1.14
xynP	32.02	36.61	1.14
*pgm*	5078.95	5724.25	1.13
yxjC	81.09	91.33	1.13
iolB	44.32	48.99	1.11
*pgk*	5422.94	5971.13	1.10
*ywlF*	2421.27	2526.84	1.04
*zwf*	2672.83	2708.68	1.01
*fbaA*	5096.63	4798.53	0.94
*gapA*	7210.27	6348.19	0.88
*pta*	2607.11	2276.82	0.87
*acoA*	62.03	53.74	0.87
*tkt*	3921.21	3212.77	0.82
*ywjH*	4166.18	3188.41	0.77
*fbp*	739.27	516.33	0.70
*alsS*	5558.12	3623.65	0.65
*alsD*	6286.45	3471.86	0.55
*pgi*	5106.18	2565.71	0.50
*gapB*	258.35	99.25	0.38
*acsA*	1836.8	450.84	0.25

^a^Detailed results are available in the ArrayExpress database [63] under accession number E-MTAB-2944.

^b^Some representative genes under direct CcpA-dependent CCR [32], and selected genes (italic) of glycolysis, pentose phosphate, and branching fermentation pathways to lactate, acetoin, and acetate, are listed in descending order of ratio (1A95/168).

### The *degU32* allele affects global CcpA-dependent CCR

**Figure 8:**
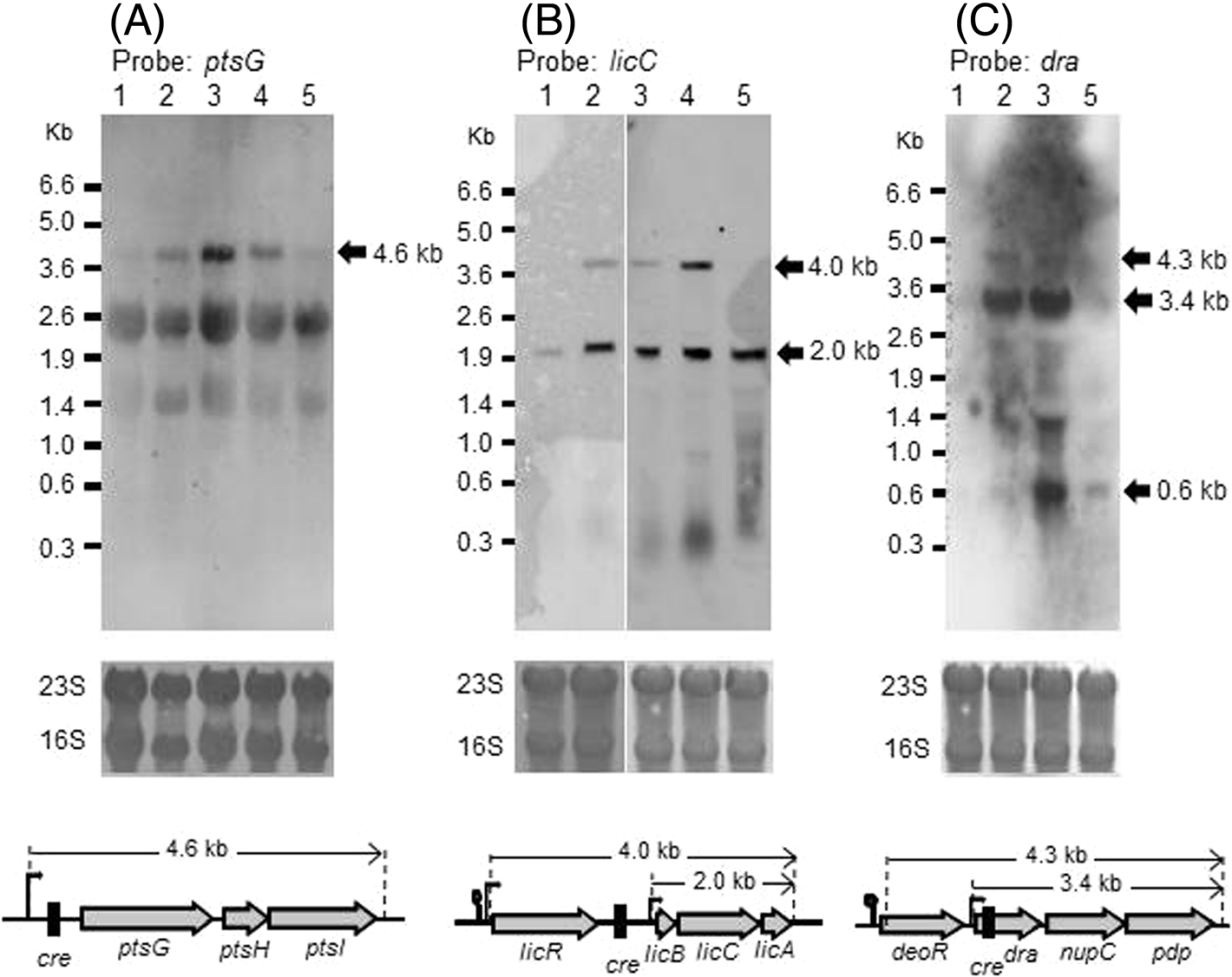
**Northern blotting analyses of transcripts containing** ***ptsG*** **,** ***licC*** **, and** ***dra.*** RNAs were prepared from 168 (lane 1), TM023 (*ccpA*::*neo*, lane 2), 1A95 (*degU32*, lane 3), TM024 (*degU32 ccpA*::*neo*, lane 4), and TM015 (*degU32*::*cat*, lane 5) cells for northern blotting analyses using probes for *ptsG* **(A)**, *licC* **(B)**, and *dra* **(C)** transcripts under higher-stringency conditions. Experiments were repeated independently at least three times with similar results and representative data are shown; rRNAs (23S and 16S) are shown on the membranes as loading controls. Genetic schemas of respective loci are shown below.

As described above, expression of *degU32* lowered intracellular FBP levels earlier, potentially reflecting an effect on global CcpA-dependent CCR. Accordingly, transcriptome analyses revealed that expression levels of some other genes that are regulated by CcpA-dependent CCR [32] were enhanced in 1A95 cells in the transition state (Table 1). Thus, *ptsGHI*, *licRBCA*, and *deoR*-*dra*-*nupC*-*pdp* were examined in specific northern blotting analyses using RNAs from transitioning cells grown in the soytone–glucose medium (Figure 8).

The *ptsGHI* operon encodes the glucose-specific PTS sugar transport system and is induced following interactions between the terminator immediately downstream of the promoter of *ptsG* and the specific antiterminator GlcT, which is activated by glucose [33]. In a previous report, a putative *cre* site located upstream of the translation initiation point of *ptsG* did not function in CCR in cells grown in LB medium [34]. However, the present northern blotting analyses of *ptsG* revealed a 4.6-kb transcript in TM023 (*ccpA*::*neo*), 1A95 (*degU32*), and in TM024 (*degU32 ccpA*::*neo*) cells, but not in 168 or TM015 (*degU32*::*cat*) cells (Figure 8A). This 4.6-kb transcript is of sufficient size to cover the entire operon, and appeared in a similar manner to the 3.7-kb transcript containing *rocG*.

The *lic* operon encodes another PTS sugar transport system for the polysaccharide lichenan [35]. A 2.0-kb transcript containing *licC* appeared in both 168 and 1A95 cells, whereas another 4.0-kb transcript was present in the strains carrying *degU32* or *ccpA*::*neo*, and then disappeared upon inactivation of *degU32* (Figure 8B). This 2.0-kb transcript may correspond with *licBCA*, and the 4.0-kb transcript may reflect *licRBCA*. Moreover, a *cre* site in the intergenic region between *licR* and *licB* [35] may be involved in CcpA-dependent CCR in 168 cells.

The *dra*-*nupC*-*pdp* operon is required for catabolism of deoxyribonucleoside [36], and although its transcription in 168 cells is strongly inhibited by glucose, it can be restored by introducing point mutations in the *cre* site within the reading frame of *dra* [37]. As shown in Figure 8C, both 3.4- and 4.3-kb transcripts containing *dra* appeared in the presence of *degU32* and *ccpA*::*neo*. A smaller 0.6-kb transcript might correspond to the monocistronic *dra* transcript, which was also dependent on *degU32* but not on *ccpA*::*neo* by unknown reason.

Taken together, the present northern blotting analyses of *ptsGHI*, *licRBCA*, and *deoR*-*dra*-*nupC*-*pdp* operons suggest that *degU32* affects *rocG* and other CcpA-dependent CCR targets.

## Discussion

Numerous studies of CCR have been performed using the *B. subtilis* strain 168 as a model of gram-positive bacteria, and the mechanisms of CcpA-dependent CCR reportedly involve formation of a regulatory *cre*-site-binding complex by CcpA and P-Ser-HPr. CcpA-dependent CCR occurs naturally in 168 cells grown in the soytone–glucose medium containing 2% glucose. In contrast, DegU-P accumulated in 1A95 cells due to the *degU32* mutation, and the presence of a novel 3.7-kb transcript containing *rocG* suggested that CcpA-dependent CCR was proactively abolished.

Most of the described genes that are regulated by the DegS–DegU system are related to flagella formation, secretion of degradative enzymes such as protease and amylase, and biofilm formation [38,39]. Although few studies reported that DegS–DegU regulated genes for intracellular metabolic enzymes, depletion of nitrogen sources reportedly increased transcription of *degU* through an interaction between TnrA and GlnA, which affected metabolism of nitrogen sources [40].

In the present study, the *degU32* mutation led to accumulation of DegU-P and elevated expression of *lctE*, which encodes a lactate dehydrogenase that may enhance lactic acid synthesis in *B. subtilis*. Under anaerobic conditions, growing cells produce ATP and consume glycolytic intermediates by alternative respiration using the electron acceptor nitrate instead of oxygen or by fermentation to produce acetone, lactic acid, and acetolactate [41]. However, under the present aerobic conditions, cells did not need to produce lactic acid for energy production. Therefore, the counter-intuitive increase in lactic acid synthesis may promote clearance of glycolytic intermediates, including FBP, and may reflect inactivation of HPrK, reduced CcpA/P-Ser-HPr activity, and CCR blockade. Schilling and colleagues [42] compared intracellular metabolites of aerobically cultured *B. subtilis* in glucose minimal medium with and without succinic acid and glutamic acid supplementation. The supplementation strongly lowered oxaloacetate synthesis but increased lactic acid synthesis significantly. Thus, the presence of organic acid in addition to glucose may induce *lctE* transcription and expression of lactate dehydrogenase, which plays an important role in the control of the intracellular redox state.

Transcriptional control of *lctE* has predominantly been examined under anaerobic conditions, and another two-component system involving ResD–ResE [43], the intracellular NAD^+^ and NADH redox sensor Rex [44], the two ECF sigma factors SigW and SigV [45], and a global regulator CodY [46] have been identified to be involved. The ResD–ResE system functions in global regulation of aerobic and anaerobic respiration and activates *resA*, *ctaA*, *qcrABC*, and *fnr* [47,48]. Transcription of *lctE* is controlled directly by Fnr, which is an integral part of the regulatory cascade required for adaptation of bacteria to low oxygen tension [49]. *B. subtilis* Fnr differs structurally from that in other bacteria such as *E. coli*, and its unique functions have not been fully characterized [50]. Nonetheless, mutations in the two Fnr-binding sites within the *lctE* promoter region disabled induction of the promoter activity [41], indicating that *lctE* may be regulated by the ResD–ResE system through Fnr. DegU-P recognizes and binds DNA carrying the relatively poorly conserved sequence motif AGAA-N11-TTCAG, and corresponding sequences are found within the promoter regions of a number of genes that are regulated by *degU*, including *wapA*, *sacB*, *sacXY*, *aprE*, *degR*, *degQ*, *srfAB*, *comC*, *comG*, *comEA*, and *mecA* [16,51,52]. In contrast, non-phosphorylated DegU reportedly binds DNA with inverted repeat sequences with the consensus ATTTA-N7-TAAAT [53], which is similar to sequences in the promoter regions of *fnr* and *lctE* (data not shown). However, further studies are required to determine whether *lctE* is regulated by non-phosphorylated *degU*. Moreover, at present we are unable to explain the mechanism how *lctE* was induced upon hyperphosphorylation of DegU.

Our transcriptome analyses revealed that *ackA* and *gntZ* were induced for 5.86-, and 4.53-fold in 1A95 cells, respectively. Especially, the induction of *ackA* seemed contradictory, because *ackA* is under positive regulation of CcpA/P-Ser-HPr and CcpA/P-Ser-Crh [31] and thus could be down regulated as FBP levels get lower. However, it is also known that *ackA* is induced by CodY in the presence of branched-chain amino acids [46]. 1A95 secretes extracellular proteases efficiently [14], and they could degrade soybean peptides in soytone–glucose medium to provide more branched-chain amino acids, which thus might trigger the CodY-dependent induction. On the other hand, *gntZ* is the last gene of the *gntRKPZ* operon for gluconate catabolism [27], which is under the control of two promoters; the one is the *gnt* promoter induced specifically in the presence of inducer gluconate and repressed with glucose via CcpA-dependent CCR [28], and the other just upstream of *gntZ* allowing its independent transcription [27]. Probably because of the absence of gluconate, the other *gnt* genes were scarcely transcribed, indicating that the *gnt* promoter was not active. Therefore, the latter promoter might be involved in the induction but is known not under CCR [27]. It might be worthwhile to investigate its specific induction upon hyperphosphorylation of DegU.

On the genetic background of the laboratory strain 168, *degU32* caused accumulation of DegU-P and production of a novel 3.7-kb transcript containing *rocG*, suggesting that CcpA-dependent CCR was proactively abolished. In our preliminary experiments in the natto starter strain NAFM5, similar phenomena occurred as in 1A95 (the results would be described elsewhere). In 168 cells, *degQ* is not efficiently transcribed due to a mutation in its cognate promoter [10]. Thus, DegU-P levels remain lower in these cells, whereas in wild-type strains such as natto starter strains, DegQ may enhance DegU phosphorylation instead of *degU32* transcription. Potential to abort CCR with hyperphosphorylation of DegU may have offered survival advantageous in many other bacteria that utilize various carbon sources in environmental competition, but could have been lost during domestication to breed the laboratory strain 168. The DegS–DegU system interacts with the ComP–ComA two-component system that is involved in natural competence. Upon increases in cell density, ComA is phosphorylated and activates DegQ [7], suggesting a shared function of DegQ in the ComP–ComA and DegS–DegU systems. However, unphosphorylated DegU is essential for the expression of the competence transcription factor encoded by *comK* [4]; and thus, hyperphosphorylation of DegU may restrict natural competence as unphosphorylated DegU decreased. Because strain 168 was selected for its high natural competence, the *degQ* promoter may have been mutated to avoid accumulation of DegU-P enhanced by DegQ following activation of the ComP–ComA system.

The present northern blotting analyses of the *rocG* locus suggested constitutive transcription from the *sivA* promoter (Figure 2B). However, this transcription is not completely terminated at the Rho-independent terminator (Figure 3C) and is usually aborted at the downstream *cre* site via the CcpA-dependent CCR roadblock mechanism [17]. Thus, upon alleviation of CCR, *rocG* may be induced immediately, leading to formation of the 3.7-kb transcript shown in 1A95 cells, which may represent a novel mode of CcpA-dependent regulation to induce the target gene. In this study, it was implied that hyperphosphorylation of DegU induced *lctE* through unknown mechanisms to decrease intracellular FBP levels earlier and thus proactively cancelled the CCR roadblock. Potentially, this mode of regulation may operate at the global level, as suggested by transcriptome (Table 1) as well as the other northern blotting analyses (Figure 8). The physiological significance of this mechanism requires further investigation.

## Conclusions

The 3.7-kb transcript covering *sivA*, *rocG*, and *rocA* may be tightly controlled under physiological conditions by a roadblock mechanism involving P-Ser-HPr/CcpA in 168 cells, while in 1A95 cells the tight control was alleviated. Accumulation of DegU-P in 1A95 cells affects central carbon metabolism involving *lctE* enhanced by unknown mechanisms, downregulates FBP levels earlier, and inactivates HPrK to allow the 3.7-kb transcription. Similar events may occur in other catabolite repressive loci.

## Methods

### Bacterial strains and media

The bacterial strains and plasmids used in this study are listed in Table 2. Strains of B. subtilis were routinely maintained on TBABG plate medium containing 3.3% tryptose blood agar base (Becton, Dickinson and Company, NJ, USA) and 0.18% glucose. To induce experimental growth, cells were precultured on TBABG plates for 16 h at 37°C, and fresh colonies were then inoculated into 30–40 ml of soytone–glucose medium containing 1.0% Bacto-soytone (Becton, Dickinson and Company), 0.5% Bacto yeast extract (Becton, Dickinson and Company), 1.0% NaCl, and 2.0% glucose (pH 7.0) in a 500-ml shaking flask at OD_600_ of 0.05, and were grown at 37°C with shaking at 150 rpm. The *Escherichia coli* strain was grown in LB medium. When required, cells were cultured in the presence of 100 μg/ml ampicillin, 0.5 μg/ml erythromycin, 50 μg/ml kanamycin, 10 μg/ml chloramphenicol, 100 μg/ml spectinomycin, or 15 μg/ml neomycin.

**Table 2:** **List of bacterial strains**

**Strain**	**Genotype**	**Source or reference**
*B. subtilis*		
168	*trpC2*	Laboratory stock
1A95	*trpC2 degU32*	[14]
WTF28	*degU::cat*	[8].
BFS3227	*trpC2 sivA*::pMutin2	NIG, Japan
ΔrocR (FW15)	*pheA1 sfp0 trpC2 rocR*::*kan*	[55]
FU402	*trpC2 ccpA*::*neo*	[57]
TM013	*trpC2 rocG*::*cat*	This study
TM014	*trpC2 degU32 rocG*::*cat*	This study
TM015	*trpC2 degU32*::*cat*	This study
TM016	*trpC2 degU32 rocR*::*kan*	This study
TM017	*trpC2 sivA*::pMutin2	This study
TM018	*trpC2 degU32 sivA*::pMutin2	This study
TM023	*trpC2 ccpA*::*neo*	This study
TM024	*trpC2 degU32 ccpA*::*neo*	This study
MY02	*trpC2 lctE*::*spc*	This study
KI004	*trpC2 degU32 lctE*::*spc*	This study
*E. coli*		
DH5α	*supE44* Δ*lacU169 hsdR17 recA1 endA1 gyrA1 gyrA96 thi*-1 *relA1*	Laboratory stock
Plasmid		
pGP958	*rocG*::*cat*	[54]

### Construction of strains

The strains TM013 and TM014 were constructed from respective parental strains 168 and 1A95 by transformation with pGP958 plasmid DNA [54] harboring a chloramphenicol resistance gene cassette inactivating *rocG*. To construct TM015, 1A95 cells were transformed with chromosomal DNA from WTF28 [8] carrying a chloramphenicol resistance gene cassette inactivating *degU32*. To construct TM016, 1A95 cells were transformed with DNA from the strain ΔrocR (FW15) [55], which harbored a kanamycin resistance gene cassette inactivating *rocR*. TM017 and TM018 strains were constructed by transformation of 168 and 1A95 cells with DNA from the strain BFS3227 (National BioResource Project; NIG, Japan; *B. subtilis*), which harbored an erythromycin resistance gene cassette inactivating *sivA* (formerly *yweA*) [56]. TM023 and TM024 strains were constructed by transformation of 168 and 1A95 cells with DNA from the strain FU402 [57], which harbored a neomycin resistance gene cassette inactivating *ccpA*.

MY02 and KI004 were constructed as follows: two DNA fragments (approximately 500-bp long) corresponding to the flanking regions upstream and downstream of *lctE* (also called *ldh*) were amplified using chromosomal DNA from 168 cells as a PCR template and the respective primers lctE-R2/lctE-R1 and lctE-F2/lctE-F1 (Table 3). Another PCR fragment containing the spectinomycin resistance gene cassette was amplified using DNA from the strain FU341 [58] and the primers lctE-spec-R and lctE-spec-F (Table 3). A mixture of the three DNA fragments was used as a template for PCR, and the primer pair lctE-R2/lctE-F1 was used to generate a recombinant fragment of the spectinomycin resistance gene sandwiched between the flanking regions upstream and downstream of *lctE*. This recombinant fragment was used to transform 168 and 1A95 cells with the spectinomycin resistance gene and inactivated *lctE* to obtain MY02 and KI004 strains, respectively.

**Table 3:** **List of oligonucleotide primers**

**Name**	**Sequence (5′ to 3′)**
lctE-F1	ttggagccaggtaaatgctt
lctE-R1	acaaaacccgctccgatta
lctE-spec-F	taatcggagcgggttttgtcaataacgctattgggag
lctE-spec-R	cagctcagtgatacctgcgactatatgctccttctggc
lctE-F2	tcgcaggtatcactgagctg
lctE-R2	gcaatgctggaccgaataat
NrocG-F	atattccagctcccgatgtg
NrocG-R-T7	taatacgactcactatagggtggtgaccataccaaagctg
NptsG-F	catcaatcgaggcaaaaaca
NptsG-R-T7	taatacgactcactatagggatcaccagctgcgtttcag
NlicC-F	gcagcaaaatttgtcgaggt
NlicC-R-T7	taatacgactcactataggggacagtcagcgttttggtca
Ndra-F	cagctttgaaaccgcataca
Ndra-R-T7	taatacgactcactatagggcggcctctaccattgtgtct
NlctE-F	ccggtcaaaacatcttacgg
NlctE-R-T7	taatacgactcactatagggagttcactgaccggcacac
PEyweA-F	aacaggaaggaattctcattgaa
PEyweA-R	atggtccactctttcgtgct

### RNA analysis

RNA samples were prepared as previously described [59] with some modifications. Briefly, cells were grown to the transition state from logarithmic and stationary phases (4 h after inoculation). Subsequently, 20-ml aliquots of the culture were centrifuged, and cell pellets were resuspended in 1 ml of LETS buffer containing 1% SDS, 100 mM LiCl, 10 mM EDTA, and 10 mM Tris–HCl (pH 7.4). After addition of 500 μl of glass beads (φ0.5 mm) and 1 ml of phenol:chloroform:isoamyl alcohol (25:24:1), cells were disrupted with vigorous shaking and were then centrifuged. Finally, purified RNA was precipitated from the upper aqueous phase using ethanol.

Northern blotting and primer extension analyses were performed as described previously [60]. Briefly, probes for northern blotting analyses were prepared using in vitro transcription with T7 RNA polymerase from template DNAs of PCR fragments that were generated using the following respective primer pairs (Table 2): *rocG*, NrocG-F/NrocG-R-T7; *sivA*, NyweA-F/NyweA-R-T7; *rocA*, NrocA-F/NrocA-R-T7; *ltcE*, NlctE-F/NlctE-R-T7; *ptsG*, NptsG-F/NptsG-R-T7; *licC*, NlicC-F/NlicC-R-T7; and *dra*, Ndra-F/Ndra-R-T7. Hybridization was performed for 16 h at 50°C (lower stringency) or 55°C (higher stringency). In primer extension experiments for the *sivA* promoter region, RNA samples were subjected to reverse transcription using the specific primer PEyweA-R (Table 2), which was 5′-end labelled with [γ-^32^P] ATP (GE Healthcare Bio-Sciences, NJ, USA) using a MEGALABEL kit (Takara Bio Inc., Otsu, Japan). Sequence ladders were produced from the same primer using dideoxy sequencing reactions, and the template PCR fragment was amplified from the 168 strain using the primers PEyweA-F and PEyweA-R (Table 3).

Transcriptome analyses were performed using a high-density tiling oligonucleotide chip as described previously [61]. Briefly, cDNAs were synthesized from RNAs and were labelled and hybridized to the oligonucleotide chip using established methods [62]. To compensate for differences in hybridization efficiency of 25-mer oligonucleotide probes on the chip, hybridization intensities of cDNAs were divided with that of total genome DNA. Normalized transcriptional signals were processed and analyzed using the In Silico Molecular Cloning program, Array Edition (In Silico Biology, Yokohama, Japan). The data are available in the ArrayExpress database [63] under accession number E-MTAB-2944.

### Measurement of metabolites

Metabolic intermediates of central carbon metabolism were determined in 168 and 1A95 cells grown on the soytone–glucose medium. At the indicated time points, 10-ml aliquots of bacterial cultures were quickly mixed into 14 ml of methanol at −40°C, and the mixture was then incubated for a further 15 min at −40°C. After centrifugation at 7,000 rpm for 5 minutes at −20°C, the supernatant was discarded and 7.5 μl of 100 mM adipic acid and 7.5 μl of 1 mM 1,4-piperazinediethanesulfonic acid were added to the remaining cell pellets as internal standards. To extract metabolites, cell pellets were disrupted with vigorous mixing in 1 ml of 75% (v/v) ethanol that was preheated to 95°C. Extracts were then evaporated under vacuum and subjected to the CE–MS analysis performed as previously described [64]. Ammonia contents of culture media were measured using an ammonia assay kit (Sigma-Aldrich, MO, USA) according to the supplier’s instructions.

## Additional file 1

Additional file 1: Figure S1. Northern blotting analyses of transcripts containing *rocB* RNAs were prepared from 168 (lane 1), 1A95 (*degU32*, lane 2), TM015 (*degU32*::*cat*, lane 3), TM016 (*degU32 rocR*::*kan*, lane 4), and TM024 (*degU32 ccpA*::*neo*, lane 5) cells*.* The *rocB-*specific probe was prepared using in vitro transcription with T7 RNA polymerase from the template DNA of PCR fragment that was generated using the primer pairs of NrocB-F (5′-aatcaggcgagtggatgttc-3′) and NrocB-R-T7 (5′-taatacgactcactatagggtattgtggagacggcttgtg-3′). rRNAs (23S and 16S) on membrane were visualized as a loading control using methylene blue staining.
