# Supplementary figures and images for: Hyperphosphorylation of DegU cancels CcpA-dependent catabolite repression of rocG in Bacillus subtilis
Source: BMC Microbiol. 2015 Feb 22;15:43. doi: 10.1186/s12866-015-0373-0 (PMC4348106; doi:10.1186/s12866-015-0373-0)

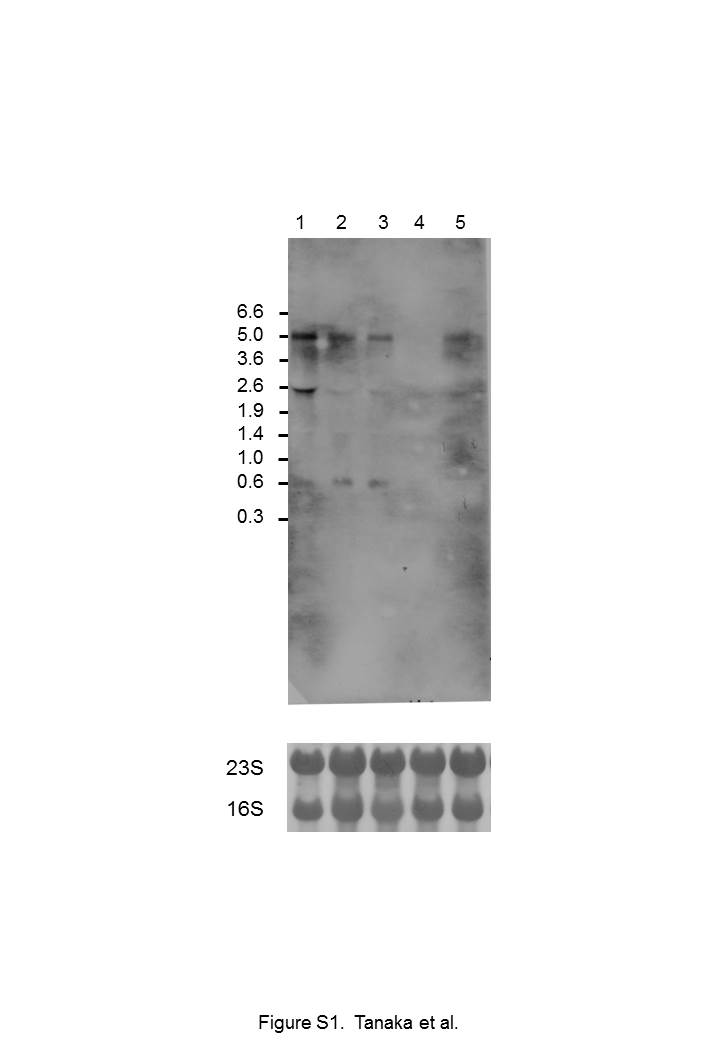

Supplement: Additional file 1: Figure S1. — Northern blotting analyses of transcripts containing rocB RNAs were prepared from 168 (lane 1), 1A95 (degU32, lane 2), TM015 (degU32::cat, lane 3), TM016 (degU32 rocR::kan, lane 4), and TM024 (degU32 ccpA::neo, lane 5) cells. The rocB-specific probe was prepared using in vitro transcription with T7 RNA polymerase from the template DNA of PCR fragment that was generated using the primer pairs of NrocB-F (5′-aatcaggcgagtggatgttc-3′) and NrocB-R-T7 (5′-taatacgactcactatagggtattgtggagacggcttgtg-3′). rRNAs (23S and 16S) on membrane were visualized as a loading control using methylene blue staining. [file 12866_2015_373_MOESM1_ESM.jpeg]
